# Effect of metabolites on the survival and biofilm formation of *Pantoea piersonii* (Basionym: *Kalamiella piersonii*) in synthetic urine media

**DOI:** 10.1186/s12866-025-04684-z

**Published:** 2026-01-20

**Authors:** Yuvarajan Subramaniyan, K. S. Megha, K. Adithyan, Rajendu R. Nair, M. Mujeeburahiman, Blessy M. Baby, Pallavi Poojarira Ganesh, Punchappady Devasya Rekha

**Affiliations:** 1 Division of Microbiology and Biotechnology, Yenepoya (Deemed to be University), Yenepoya Research Centre, University Road, Deralakatte, Mangalore, 575018 India; 2 Department of Urology, Yenepoya Medical College and Hospital, Yenepoya (Deemed to be University), University Road, Deralakatte, Mangalore, 575018 India

**Keywords:** Urinary tract infection, Kalamiella piersonii, Pantoea piersonii, Polymicrobial biofilm, Virulence, Urological diseases

## Abstract

*Pantoea piersonii* (Basionym: *Kalamiella piersonii*) is an opportunistic pathogen capable of forming strong biofilms and infecting diverse anatomical sites. In this study, we investigated the growth, biofilm forming ability and ureolytic activity of *P. piersonii* under different growth conditions using synthetic urine to test its adaptability under different urinary conditions. We used *P. piersonii* strain YU22, isolated from the urine of a patient with kidney stone disease, and cultured under different urea concentrations (10 mM − 420 mM) to evaluate urea tolerance. Glucose, creatinine, and albumin were supplemented in synthetic urine to simulate the glycosuria, creatinuria, and albuminuria. Additionally, the effect of urinary pH variation was examined by adjusting the media to pH 5, pH 7, and pH 8. To investigate the ability of *P. piersonii* to co-exist with other key uropathogenic bacteria, we used a uropathogenic *Escherichia coli*, in a co-culture experiment. Under all the tested conditions, the growth, biofilm formation, and ureolytic activity were quantified using standard methods. Expression of urea carboxylase and allophanate hydrolase, in the selected conditions was analyzed using qRT-PCR. *P. piersonii* was able to tolerate urea at all the tested concentrations, showing a significant increase in growth (OD_600_) with increasing concentrations of urea from 0.19 ± 0.01 (10 mM) to 0.59 ± 0.01 (420 mM) (*p* < 0.01). Biofilm formation measured by crystal violet staining method also showed significant increase with OD_590_ values from 10 mM (0.55 ± 0.03) to 420 mM urea (1.74 ± 0.05) (*p* < 0.01). Among the nutritional supplements the highest growth and biofilm formation was observed in glucose, followed by creatinine and albumin. The combination of glucose with creatinine resulted in 25% and 30% increase in growth, respectively, compared to media containing glucose or creatinine separately. pH 7 favored growth and biofilm formation in all the tested conditions, except in the presence of glucose. Growth response and ureolytic activity varied significantly with changes in the nutritional composition and pH of the media (*p* < 0.05). In dual-species biofilm environment, *P. piersonii* dominated over *E. coli* under the tested growth conditions, suggesting a competitive advantage in the urinary environment that may influence community structure in mixed-species biofilms. These results demonstrate that the survival mechanism and ureolytic activity of *P. piersonii* are complex with the ability to adapt to varying environmental conditions in response to nutrient availability to enable survival under extreme conditions. However, these preliminary findings require validation through further investigations.

**Supplementary Information:**

The online version contains supplementary material available at 10.1186/s12866-025-04684-z.

## Introduction

*Pantoea piersonii* (*P. piersonii*) *(Basionym: Kalamiella piersonii)* is a Gram-negative bacterium belonging to the family Erwiniaceae, first identified from the International Space Station [1]. Subsequent evidences support that *P. piersonii* is an opportunistic pathogen having the ability to be associated with urinary tract, pulmonary, and gastrointestinal infections, and soft tissue infections, bacteremia across all age groups, often complicated by antimicrobial resistance (AMR), suggesting its potential role in the silent AMR epidemic [2–6]. Genomic evidences have revealed multiple virulence traits in *P. piersonii*, including adhesion, invasion, colonization, siderophore production, swarming behavior, and biofilm formation [2, 5]. In vitro experiments using one of the strains (YU22) demonstrated the biofilm-forming ability and ureolytic activities, supporting its survival in the nutrient-limited urinary tract environment [2, 7]. Biofilm formation is one of the major virulence factors in uropathogens, and it protects against antibiotics and environmental stresses, enabling bacterial persistence and immune evasion [8–11]. Biofilm formation is enabled by various factors, including swarming motility to facilitate surface colonization, initial attachment and microcolony formation by promoting coordinated movement. It also assists uropathogens in ascending toward the bladder, contributing to upper urinary tract infections (UTI) [12, 13]. Uropathogens also possess other virulence traits, such as siderophore production, which may support biofilm formation by regulating surface-associated activities and stabilizing the polysaccharide matrix, while iron restriction reduces surface hydrophobicity and alters surface protein composition, hindering biofilm development [14]. Siderophore is required to efficiently acquire iron from the environment, which supports their growth and contributes to the severity of infection [15].

Uropathogens colonizing the urinary tract generally have the ability to either utilize or tolerate the high concentrations of urea. In many uropathogens, urease is a key enzyme that hydrolyzes urea into ammonia and CO_2_, thereby providing essential nutritional support for bacterial growth and survival [16]. Urease activity also plays a critical role in pH homeostasis and survival under weak acid stress, enabling pathogens to counteract intracellular acidification in urea-rich environments. In *P. piersonii*, an alternative ureolytic pathway has been reported, namely urea amidolyase [UAL], involving a multifunctional enzyme system composed of urea carboxylase (UC) and allophanate hydrolase (AH), which effectively degrades urea and changes the pH in nutrient-limited conditions [2, 7]. These virulence traits can aid in the severity of infection and serve as critical survival mechanism under urine infection associated conditions.

The urinary metabolite composition varies under renal and lifestyle-related metabolic diseases, that may lead to changes in the levels of glucose, creatinine, albumin, and other metabolites, which fluctuate substantially as kidney function declines and glomerular filtration rate decreases [17]. Glycosuria becomes evident when urinary glucose exceeds 5 mM, with > 16.5 mM and > 33.3 mM indicating moderate to severe glycosuria [18–20]. Urinary creatinine is typically present at approximately 23 mM in women and 27 mM in men, but its concentration can vary widely in different diseases, ranging from < 10 mM to > 100 mM [21, 22]. Albuminuria is clinically important due to its association with vascular dysfunction, renal injury, diabetes, and other kidney disorders, and is defined as elevated urinary albumin excretion ranging from > 20 mg/L (microalbuminuria) to > 200 mg/L (macroalbuminuria) [23]. Urine pH varies under a wide range of physiological, dietary, and pathological conditions. In diabetic patients and individuals with chronic kidney disease the urine pH can range from 5.0 to 6.5, whereas, those with other renal metabolic disorders or alkalosis often present with pH levels exceeding 7.5 [24, 25]. Nutritional availability and urine pH can significantly influence the colonization of uropathogens. For example, acidic conditions have been shown to enhance adherence, motility, and biofilm formation in uropathogenic *Escherichia coli* (*E. coli*) and *Klebsiella pneumoniae* (*K. pneumoniae*) [26, 27].

Polymicrobial interactions among uropathogens are commonly reported from the urinary environment, and are facilitated by interspecies cooperation, including nutrient sharing, quorum-sensing, coordinated gene expression, and metabolic exchange [28, 29]. Many reports have shown that biofilms involved in catheter-associated UTI are predominantly polymicrobial communities, commonly involving *E. coli* along with *Pseudomonas aeruginosa (P. aeruginosa)*, *K. pneumoniae*, and *Proteus mirabilis* [30–32]. Nutritional changes in the urinary environment can provide selective advantages to the coexisting uropathogens, where competitive interactions may lead to dominance of one species, while cooperative interactions may support a stable equilibrium within the polymicrobial community [30, 33].

Based on this background, we investigated the survival ability of *P. piersonii* strain YU22 in the urinary environment by challenging it with elevated levels of glucose, creatinine, and albumin and pH-variable conditions in vitro using synthetic urine media. We further assessed the response of its ureolytic enzyme system under these nutritional conditions to determine whether its pH-regulating capacity parallels that of classical urease in maintaining pH homeostasis. In addition, we examined its co-colonization potential with uropathogenic *E. coli* to evaluate whether metabolic cooperation or competitive interactions facilitate its survival within polymicrobial infections.

## Methodology

### Ethical statement

The study was conducted and approved in compliance with the ethical requirements of the Yenepoya Ethics Committee-1 (Protocol no. YEC-1/2021/065) and was performed with the institutional and National Ethical Guidelines for Biomedical and Health Research involving Human Participants (2017), in accordance with the Declaration of Helsinki (2024). The requirement for informed consent was waived by the Yenepoya Ethics Committee-1 (Protocol no. YEC-1/2021/065) whenever anonymized samples were used.

### Bacterial strain and growth conditions

*P. piersonii* strain YU22 (MCC3118), previously isolated from the urine of a patient with struvite kidney stone, was used in the study [2]. For the dual-species culture, uropathogenic *E. coli* YPD-Y50E, previously isolated from a case of emphysematous pyelonephritis, was used (GenBank accession number for the 16S rRNA gene: OR646680). The bacteria were revived from the − 80 °C freezer and cultured on nutrient agar media (HiMedia, India). For inoculum preparation single colony was inoculated into the nutrient broth and incubated at 37 °C overnight under agitation (120 rpm). For all the experiments, an initial cell density of 10^5^ CFU/mL and incubation temperature of 37 °C were maintained unless specified. For dual species culture half of the initial inoculum volume of each bacteria was used to maintain uniform cell density.

### Growth media

For urea tolerance experiments, we used M9U media supplemented with a range of urea concentrations (10–420 mM). The composition of M9U media is given in Table S1. Further synthetic urine media was used to study the effect of glucose, creatinine, and albumin on the bacterial growth and biofilm. Synthetic urine was prepared as described earlier with slight modification [19, 34]. The composition included (in g/L): CaCl_2_. 2H_2_O (0.651); MgCl_2_. 6H_2_O (0.651); NaCl (4.6); Na_2_SO_4_ (2.3); KH_2_PO_4_ (2.8); KCl (1.6); sodium citrate (0.65); sodium oxalate (0.02); glucose (0.14); albumin (0.02); urea (25.0); creatinine (2.5). Synthetic urine was supplemented with glucose (19 mM), creatinine (53 mM), and albumin (300 mg/L) to reflect the glycosuria, creatininuria, and albuminuria. Further, to determine how pH variations within the urinary environment influence pH homeostasis and biofilm formation in YU22, synthetic urine was adjusted to pH 5.0, 7.0, and 8.0 and tested in the presence of glucose, creatinine, and albumin.

Additional concentrations of glucose (5 mM and 35 mM) and creatinine (10 mM and 100 mM) were supplemented to synthetic urine to assess their combined effects on growth, biofilm, and ureolysis in experimental assays.

### Experimental growth conditions

For the experiments, bacterial cells were inoculated into 50 mL borosilicate glass tubes containing 10 mL of medium. The contents were incubated with agitation (120 rpm) for 24 h. For biofilm development, prior to the incubation, 200 µL samples were transferred to a 96-well plate and incubated separately under static conditions. For evaluating time-dependent growth and biofilm formation, selected urea concentrations of 160 mM and 420 mM, which fall within the normal physiological range of urea in human urine, were used and incubated for 72 h in separate 96-well plates for harvesting at different intervals.

### Assessment of planktonic growth and biofilm formation

After the incubation period, to assess planktonic growth, absorbance at 600 nm (A_600_) was measured using a microplate reader (FLUOstar^®^ Omega, BMG Labtech, Germany). Subsequently, the planktonic cells were removed, and biofilm was quantified by the crystal violet staining method as described previously [35]. Briefly, the adherent biofilm was gently washed with phosphate-buffered saline (PBS) to eliminate loosely bound cells, fixed with methanol (95%) and stained with crystal violet (0.1% aq, w/v) for 10–15 min. The excess stain was removed by PBS wash, and the stain was solubilized using acetic acid (33%). Biofilm intensity was quantified by measuring the absorbance at 590 nm (A_590_) using a microplate reader.

To enumerate the bacterial biomass standard plate count method was used. For this, the biofilms were developed separately as described above, and the planktonic cells were carefully removed. The biofilm was washed and disrupted by gentle scraping, and the cells were released by flushing and mixing with a pipette tip. The biofilm contents were re-suspended in PBS, serially diluted, and plated on nutrient agar media to determine biofilm biomass (CFU/mL). The planktonic cells were also subjected to a standard plate count after serial dilution.

For visualisation, biofilm was developed on sterile glass coupons (1 cm^2^) in 24-well plates containing 2 mL of synthetic urine supplemented with glucose, creatinine, and albumin for 24 h. The coupons were removed, washed and fixed with methanol, biofilm cells were stained with acridine orange dye (0.3% aq, w/v) in dark. The stained biofilm was observed and imaged using the green (FITC/GFP) fluorescence channel of the ZOE™ Fluorescent Cell Imager (ZoE BioRad), which uses a 470–495 nm excitation LED and a 510–545 nm emission filter. A representative three-dimensional (3D) image reconstruction was carried out with the following parameters, including grid size of 256, with smoothing set to 0.0, perspective to 0.1, lighting to 0.2, and a Z-scale of 0.10, was used to construct the 3D image using ImageJ software (version 1.8.0_172).

### Assessment of ureolytic activity

To assess the ureolytic activity under different growth conditions, pH of the culture was directly measured using a digital pH meter (Systronics µ pH System 361). Further, NH_4_-N levels were quantified as a measure of ureolysis for confirmation of the activity, using a previously described method [2, 36]. Briefly, 40 µL of cell-free supernatant, 80 µL of a sodium cocktail solution, and 80 µL of 10% sodium hypochlorite were added to 96-well microplate. The contents were incubated for 40 min and the absorbance was measured at 650 nm using a microplate reader, and NH_4_-N was quantified using (NH_4_)_2_SO_4_ as the standard. Appropriate cell-free blanks were used to measure the accurate changes in ureolytic activity and ammonia nitrogen quantification.

To validate this, the expression of *UC* and *AH* genes of *P. piersonii* was evaluated using synthetic urine media supplemented with glucose, creatinine, and albumin by quantitative real-time PCR (qRT-PCR). Total RNA was extracted from 3 mL of the 24 h cultures, using RNAiso plus Trizol reagent (Cat# 9108/9109 TaKaRa) following the manufacturer’s protocol. The quality and quantity of the extracted RNA were measured using a microvolume spectrophotometer (Colibri, Berthold Technologies GmbH & Co. KG). The cDNA was synthesized from total RNA using the Prime Script 1 st strand cDNA synthesis kit (Cat#6110A, TaKaRa) according to the manufacturer’s protocol in the SureCyc`ler 8800 PCR system (Agilent Technologies). The qRT-PCR was performed using TB Green^®^ Premix Ex Taq™ II master mix (Cat#RR820A, Tli RNase H Plus, TaKaRa) in a CFX96 q-PCR instrument (BioRad). The 16 S rRNA gene was used as a housekeeping gene for normalization of the data. Relative gene expression was calculated using the 2^−ΔΔCT^ method and expressed as fold-change compared to the control. The details of the primers used and PCR conditions are listed in Table S2.

### Measurement of swarming motility and siderophore production

Swarming motility and siderophore production were measured in synthetic urine supplemented with glucose, creatinine, and albumin. To check swarming motility, 0.4% agar was autoclaved and mixed with sterile synthetic urine base and specific nutritional inputs were aseptically supplemented after autoclaving. The prepared synthetic urine medium supplemented with metabolites was poured into petri plates and kept upright position. From the overnight cultures grown on nutrient agar, single, pure colonies of uniform size were picked and point-inoculated onto the semisolid agar plates. The plates were incubated in an upright position at 37 °C for 96 h to allow colony swarming. The colony diameters were measured by taking at least three measurements along different swarming axes to account for irregular swarming morphologies. A few representative photographs were taken using a mobile phone camera (Mi 10i, Xiaomi; 108 MP) to illustrate the swarming pattern.

For quantification of siderophore production, experiments were conducted in synthetic urine media as mentioned in the earlier section and measured using the chrome azurol S (CAS) assay. After 24 h incubation cell-free supernatants from 1 mL of cultures were mixed with an equal volume of CAS reagent. The contents were incubated at 25 °C for 15 min, and the absorbance was measured at 630 nm. Siderophore production was quantified from the absorbance values of the cell-free blank and the cell-free supernatant of test samples, as described previously [37].

### Assessment of dual species biofilm

To evaluate the interaction of *P. piersonii* with other common uropathogens, it was co-inoculated with a uropathogenic *E. coli* (YPD-Y50E). The optimized inoculum volume was selected to maintain a uniform cell density between the two strains and incubated at 37 °C under static conditions for 24 h. After incubation, the planktonic growth and biofilm formation were assessed as described in earlier sections. To quantify biofilm biomass under dual species culture conditions, cells were enumerated using the standard plate count method on MacConkey agar. This medium enables differentiation between *P. piersonii* and *E. coli* based on lactose fermentation. *E. coli*, a lactose-fermenting bacterium, forms characteristic pink colonies, whereas *P. piersonii*, a non-lactose-fermenting strain, produces pale or colorless colonies. This distinction allowed accurate enumeration of each species within the mixed biofilm. The planktonic and biofilm cell biomass were represented in terms of CFU.

### Statistical analysis

All experiments were conducted at least in triplicates, and the data are presented as the mean ± standard deviation. Statistical significance was assessed between the groups by one-way and two-way analysis of variance (ANOVA). The significance level (*p*-value) was determined by Tukey’s multiple comparisons and Dunnett’s test. All the statistical analyses were performed using Microsoft Excel (Microsoft Office 365 version 16.0) and GraphPad Prism (Version 8.0.2).

## Results

### Urea tolerance is associated with increased biofilm formation in *P. piersoni*

**Fig. 1:**
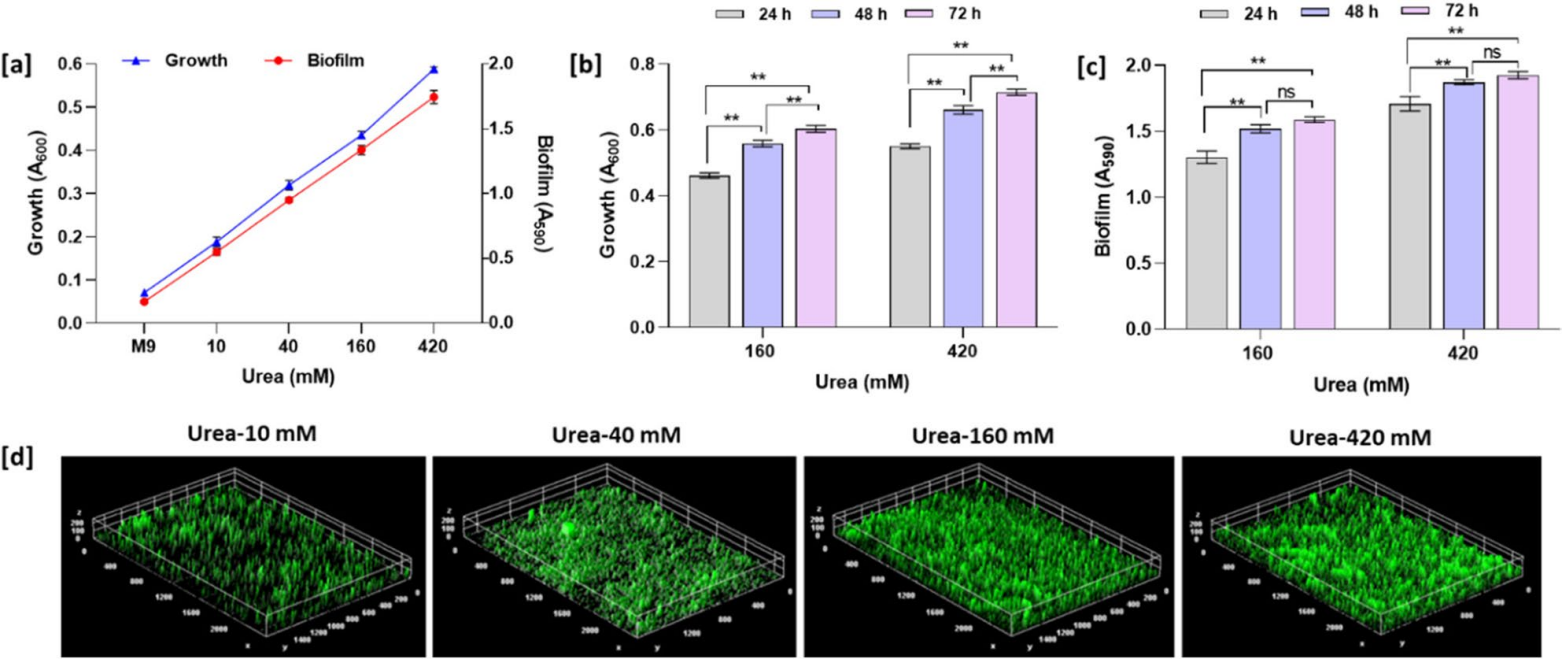
Effect of urea concentrations on growth and biofilm formation of *P. piersonii*. Growth and corresponding biofilm formation in the M9U media supplemented with 10 to 420 mM urea (**a**), growth and biofilm at different time points in M9U media with 160 and 420 mM urea levels (**b**, **c**). Growth was measured as absorbance at 600 nm (A_600_), and biofilm was quantified using 0.1% crystal violet staining, as 590 nm (A_590_). The data presented are corrected with the respective cell free blanks. Visualization of acridine orange stained biofilms developed on glass coupons under different urea levels in M9U (**d**). Data are presented as mean ± SD (*n* = 3). ns and ** represent the significance of the difference at *p* > 0.05 and *p* < 0.01, respectively. ns; not significant

The urea tolerance of *P. piersonii* was evaluated in M9U medium using urea as the sole carbon and nitrogen source at concentrations ranging from 10 to 420 mM. We observed a significant increase in the growth (A_600_) of *P. piersonii* YU22 with increasing urea concentration from 0.19 ± 0.01 at 10 mM to 0.59 ± 0.01 at 420 mM (*p* < 0.01) (Fig. 1a). Similarly, biofilm formation also showed an increasing trend with increasing urea concentrations, with biofilm intensity (A_590_) ranging from 0.55 ± 0.03 at 10 mM to 1.74 ± 0.05 at 420 mM (*p* < 0.01).

Further, we evaluated the longer-term survival of the bacterium in M9U medium containing 160 mM and 420 mM urea for 72 h. The results showed that the bacteria could survive for 72 h period, with growth (A_600_) increasing from 0.46 ± 0.01 (24 h) to 0.60 ± 0.01 (72 h) at 160 mM, and from 0.55 ± 0.01 (24 h) to 0.71 ± 0.01 (72 h) at 420 mM (Fig. 1b). Biofilm formation also increased over the incubation time as shown in Fig. 1c, however, no significant increase was observed between 48 h and 72 h at both the concentrations (*p* > 0.05).

The biofilm matrix visualised using fluorescence staining corresponded with the quantitative results (Fig. 1 d).

### Growth and biofilm formation in synthetic urine supplemented with glucose, creatinine, and albumin

**Fig. 2:**
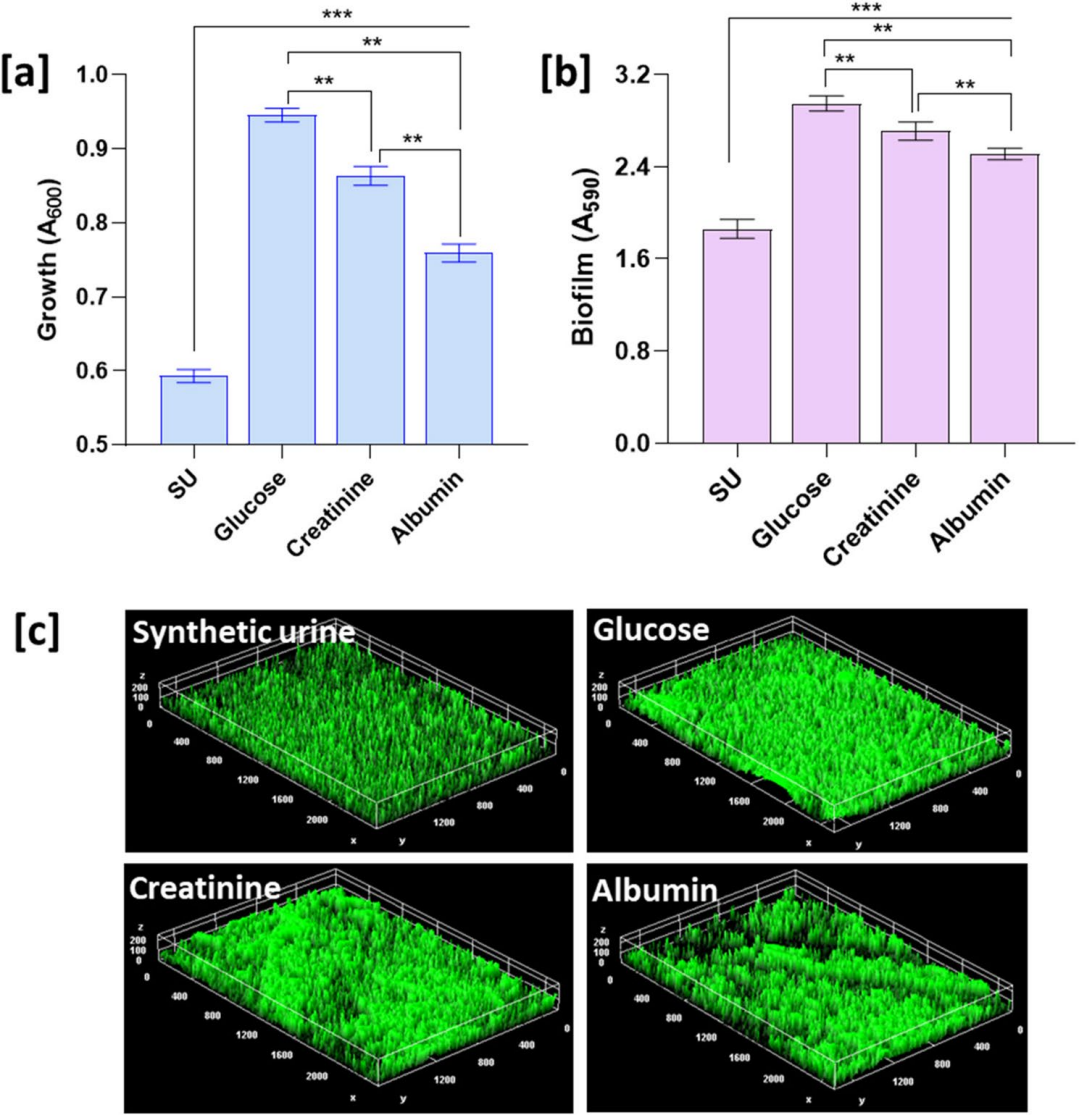
Effect of glucose, creatinine, and albumin supplementation to synthetic urine (SU) media on growth and biofilm formation of *P. piersonii*. Growth was measured as absorbance at 600 nm (A_600_) (**a**), and biofilm was quantified using crystal violet staining, measured as 590 nm (A_590_) (**b**). Representative images of acridine orange stained biofilms developed on glass coupons for illustration (**c**). Data are presented as mean ± SD (*n* = 3). ** and *** represent the significance of the difference at *p* < 0.01, and *p* < 0.001

The impact of glucose, creatinine, and albumin on growth and biofilm formation of *P. piersonii* assessed in synthetic urine media are shown in Fig. 2a and b. Among the tested conditions, glucose supported the highest growth with A_600_ values of 0.95 ± 0.01, followed by creatinine (0.86 ± 0.01) and albumin (0.76 ± 0.01). Biofilm formation was similarly influenced, with significantly higher intensities observed in glucose and creatinine supplemented conditions compared to albumin (*p* < 0.01). The biofilm matrix visualization showed denser compact biofilm matrix in glucose and creatinine compared to the control and albumin supplemented synthetic urine medium (Fig. 2c)

### Ureolytic activity of *P. piersonii* in response to nutritional supplementation

**Fig. 3:**
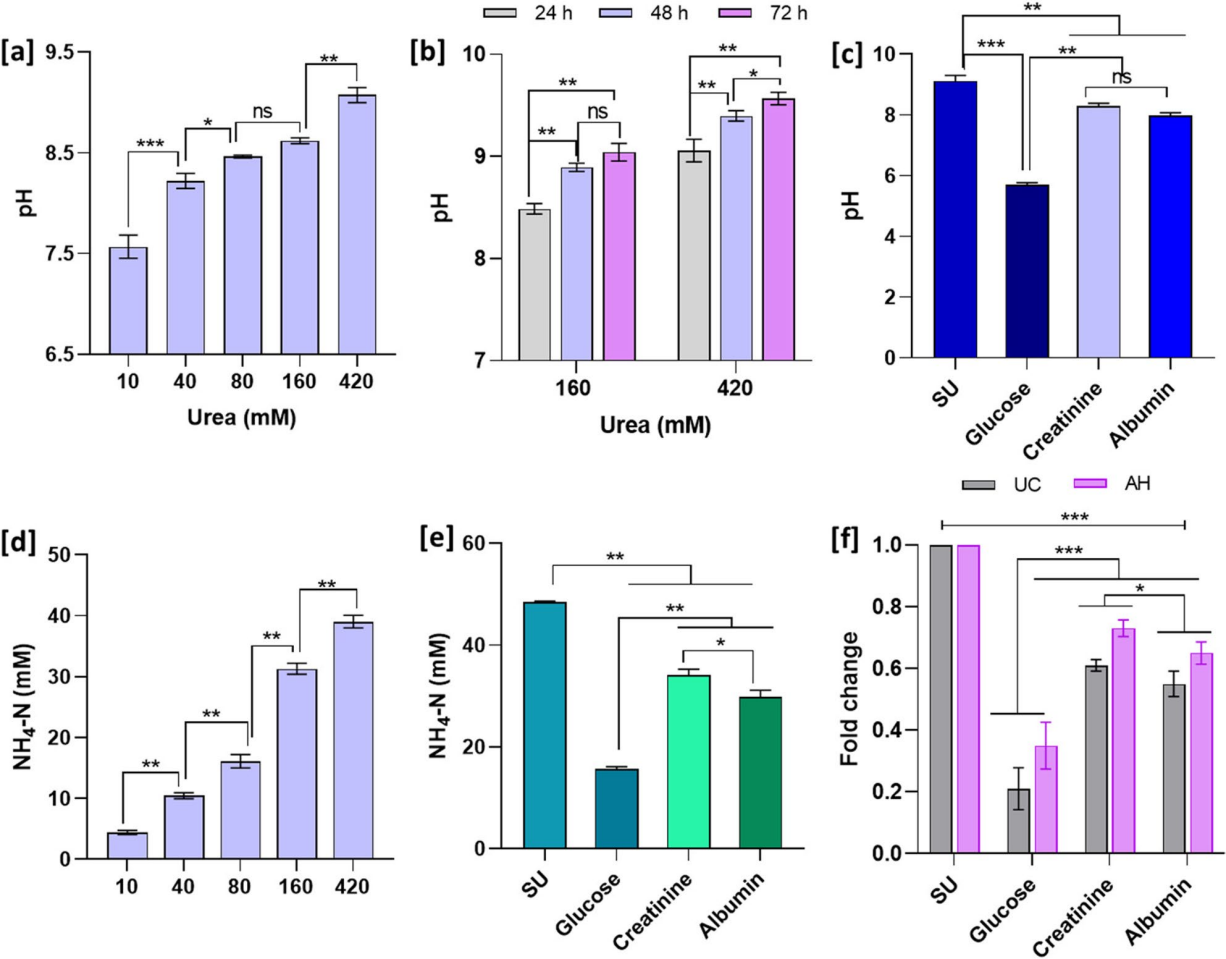
Effect of urea, glucose, creatinine and albumin on the ureolytic activity of *P. piersonii* at 24 h growth. The changes in pH levels due to bacterial ureolytic activity under different urea levels in M9U media at 24 h (**a**), time-dependent pH variations at 160 and 420 mM urea over 72 h (**b**). The influence of glucose, creatinine, and albumin on media pH (**c**). NH_4_-N levels quantified under varying urea concentrations (**d**) and in the presence of glucose, creatinine, and albumin (**e**). The relative fold-changes in the expression of *UC* and *AH* genes after 24 h growth under different growth conditions, measured using qRT-PCR (**f**). Relative gene expression was calculated using the 2^−ΔΔCT^ method and expressed as fold-change compared to the control. Data represent mean ± SD (*n* = 3). ns, *, **, and *** represent the significance of the difference at *p* > 0.05, *p* < 0.05, *p* < 0.01 and, *p* < 0.001 respectively. ns; not significant

Initially, ureolytic activity was evaluated by measuring the pH changes as an indirect method. A significant increase in the media pH was recorded with increasing urea concentrations in *P. piersonii* cultures, from an initial pH of 6.8 to > 8.0 (Fig. 3a). Similarly, over the 72 h of incubation an increase in pH was observed in both tested urea concentrations (Fig. 3b). The media pH shifted to alkaline range in all the tested nutritional conditions except in glucose where the pH was in acidic range (pH 5.7 ± 0.07) (Fig. 3c).

The ureolytic activity was further examined by quantifying NH_4_-N levels, which ranged from 4.63 ± 0.09 mM to 39.06 ± 1.03 mM in M9U media containing 10 mM and 420 mM urea respectively with significant increase (*p* < 0.01) (Fig. 3 d). In M9U media with 160 mM and 420 mM urea did not show any significant differences in the NH_4_-N levels between 48 h to 72 h of growth (*p* > 0.05) (Fig. S1). 

We also evaluated whether glucose, creatinine, and albumin modulated the ureolytic activity of *P. piersonii*. Interestingly, glucose, creatinine, and albumin supplemented synthetic urine medium significantly affected the ureolytic activity (*p* < 0.05) (Fig. 3e). The NH_4_-N levels in glucose supplemented synthetic urine media was significantly lower than all other experimental groups (*p* < 0.01). These findings were further aligned with expression patterns of the *UC* and *AH* genes, confirming the impact of the metabolites (Fig. 3f).

### Influence of glucose, creatinine, and albumin on swarming and siderophore production

**Fig. 4:**
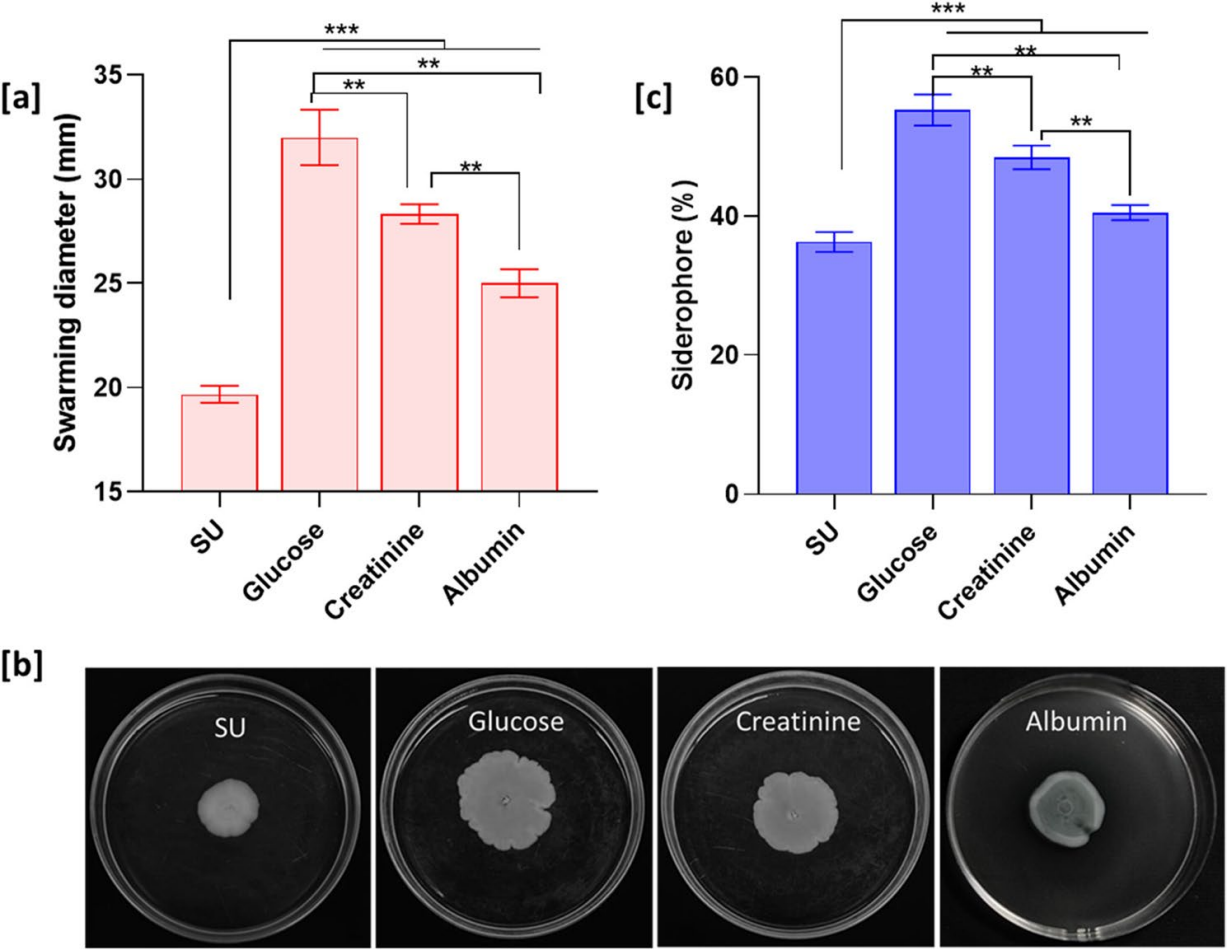
Swarming motility and siderophore production in *P. piersonii*. Quantitative analysis of swarming motility on semisolid agar (0.4%) plates prepared using synthetic urine (SU) and supplemented with glucose, creatinine, and albumin at 96 h incubation (**a**). Representative soft agar plates showing the swarming patterns (**b**). The percentage of siderophore production in YU22 quantified using the chrome azurol S reagent method at 24 h (**c**). Data points are mean ± SD (*n* = 3). ** and *** represent the significance of the difference at *p* < 0.01 and *p* < 0.001, respectively .

The influence of nutritional conditions on the swarming motility is shown in the Fig. 4a, and 4b. The swarming diameter in the synthetic urine control group was 20 ± 0.41 mm. Among the metabolite supplemented groups, the highest swarming was observed in glucose (32 ± 1.4 mm), followed by creatinine (28 ± 0.5 mm) and albumin (25 ± 0.7 mm). The swarming morphology showed slight differences among the conditions, specifically, glucose and creatinine exhibited irregular colonies with circular wave-like structure and undulate margins, whereas albumin showed smoother margins.

Siderophore production significantly varied among the growth conditions (*p* < 0.01) (Fig. 4c). Significantly higher siderophore production was seen in glucose supplementation (55.3 ± 2.2%), followed by creatinine (48.5 ± 1.7%) and albumin (40.5 ± 1.1%).

### Combined effect of glucose and creatinine on growth and biofilm formation of *P. piersonii*

**Fig. 5:**
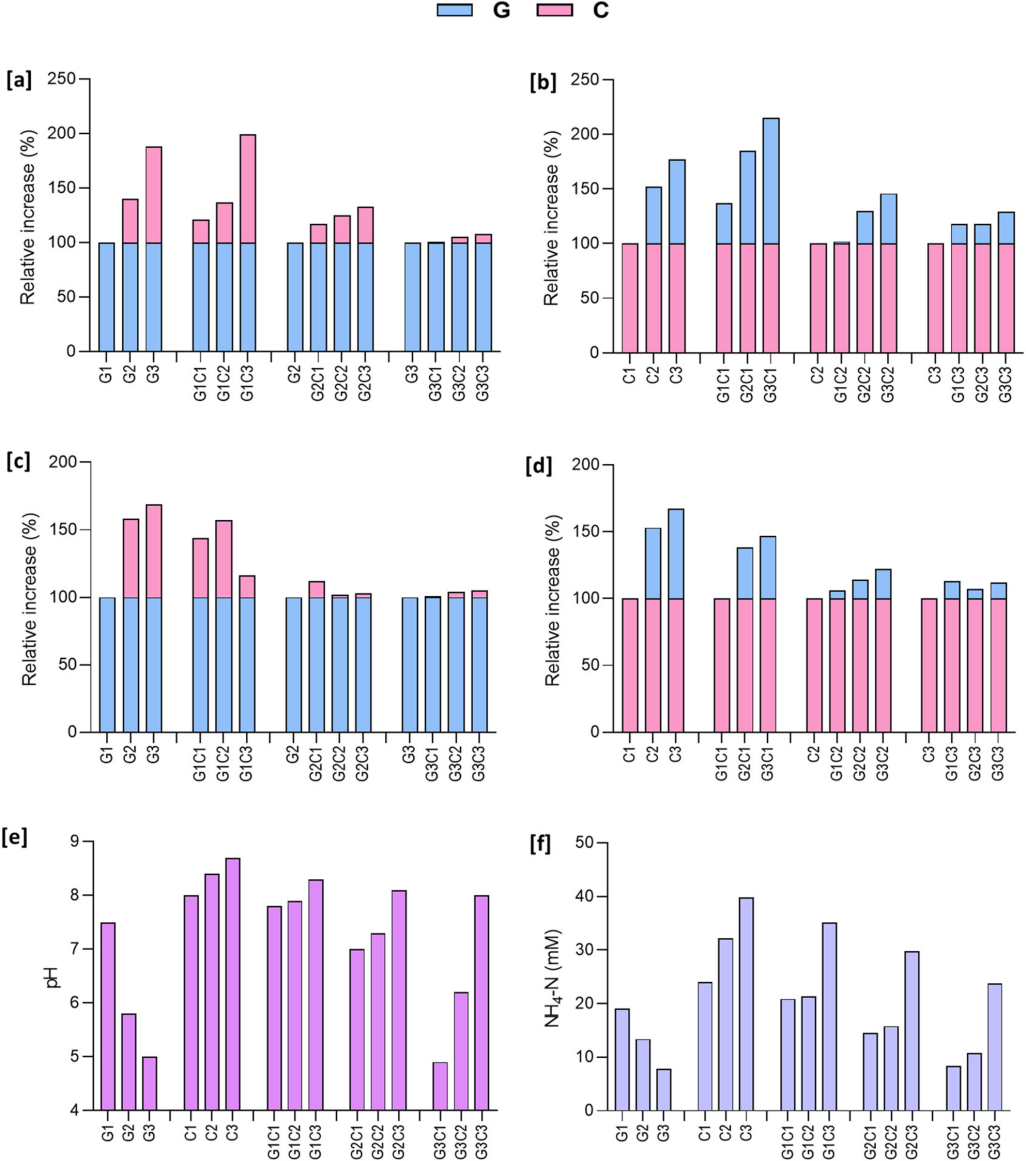
Influence of glucose and creatinine, and their combinations at varying concentrations on growth, biofilm formation, and ureolytic activity of *P. piersonii* at 24 h. The tested glucose levels are 5 mM (G1), 19 mM (G2), 35 mM (G3) and creatinine levels are 10 mM (C1), 53 mM (C2), 100 mM (C3) in synthetic urine. Relative increase in growth compared to the baseline glucose (**a**) and compared to the baseline creatinine (**b**). Relative increase in biofilm formation compared to the baseline glucose (**c**), and creatinine (**d**), pH changes (**e**), and NH_4_-N production (**f**) under different treatment groups.

We investigated how glucose and creatinine, and their combinations at varying concentrations influence the growth, biofilm formation, and ureolytic activity of *P. piersonii*. The growth showed a significant increase with an increase in the glucose and creatinine concentration (*p* < 0.01), with variation in responses among the tested combinations. The percentage differences in growth and biofilm responses for the combination relative to the individual supplements are presented in Fig. 5 a-d. Addition of creatinine to lower concentration of glucose (5 mM) in synthetic urine showed higher increase in growth with increasing creatinine concentrations compared to their respective individual concentrations (Fig. 5 a). In the combination groups, glucose 5 mM (G1) with 100 mM creatinine (C3) showed the highest growth that was 99% more than G1 group.

The media pH changes due to the ureolytic activity showed interesting results (Fig. 5e). Increase in glucose concertation decreased the pH while, creatinine alone increased the media pH. However, the creatinine combination with glucose increased the pH at the tested higher concentration of creatinine (C3) compared to respective glucose concentrations. The same effect was observed in the NH_4_-N levels (Fig. 5f). Albumin increased the growth and biofilm formation in combination with creatinine and glucose which were significantly higher than the respective single components. The pH was also increased in all conditions and maintained alkaline pH conditions (Table S3).

### pH-dependent modulation of biofilm formation and ureolysis by *P. piersonii* under nutritional conditions

**Fig. 6:**
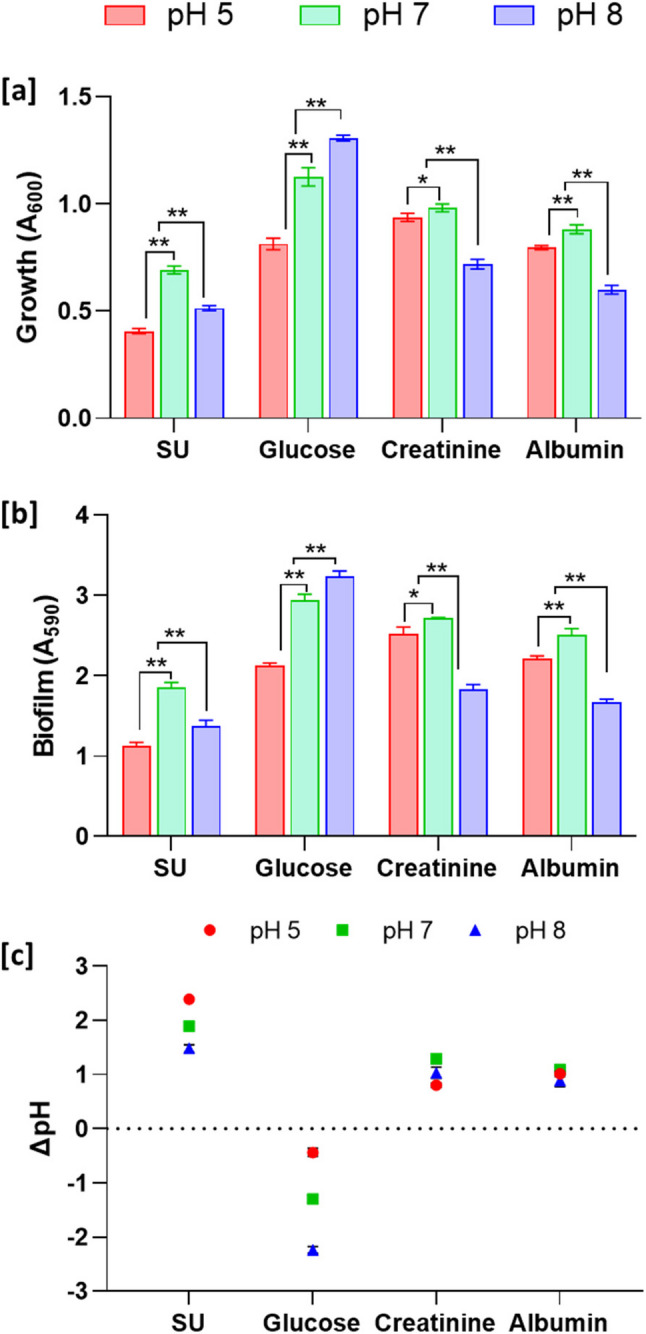
Influence of pH on growth, biofilm formation and ureolytic activity in *P. piersonii* under different nutritional conditions at 24 h. Growth measured as A_600_ (**a**), biofilm intensity measured by 0.1% crystal violet staining using absorbance A_590_ nm (**b**). Changes in media pH were measured as ΔpH changes (Final pH-Initial pH) (**c**). Data represent mean ± SD (*n* = 3). * and ** represent the significance of the difference at *p* < 0.05, and *p* < 0.01, respectively.

The effect of synthetic urine pH supplemented with glucose, creatinine, and albumin showed significant differences in growth and biofilm formation among the tested pH groups (*p* < 0.01) (Fig. 6a and b). Under control condition, the highest growth and biofilm formation were observed at pH 7. In glucose significantly higher growth and biofilm formation was observed at pH 8 compared to pH 7 and pH 5. In contrast, under the creatinine, and albumin, both higher and lower pH reduced the growth and biofilm formation compared to pH 7. The representative visual illustration of the biofilm matrix under these conditions is shown in Fig. S2.

 To understand whether *P. piersonii* uses ureolytic activity to alter the prevailing media pH, the enzyme activity was measured based on the ammonia release under different nutritional conditions. Interestingly, ureolytic activity patterns differed from those observed for growth and biofilm formation. Under control condition in the synthetic urine media significantly higher ammonia production was evident at pH 5, followed by pH 7, and with very low activity at alkaline pH (Fig. S2). Under creatinine and albumin supplemented conditions, maximal NH_4_-N liberation was also observed at neutral pH, followed by alkaline pH, with comparatively lower production under acidic conditions (*p* < 0.001). In contrast, glucose supplementation led to increased ammonia release under alkaline pH, followed by neutral and acidic pH. The pH changes under these conditions are given in Fig. 6c. In control group, the highest difference in pH at 24 h was observed in the group where the initial media was pH 5. The pH changes under glucose showed the highest difference at pH 8 (−2.24), followed by pH 7 (−1.30), maintaining acidic pH conditions. In creatinine and albumin, the pH differences were between + 0.80 (creatinine, pH 5) to + 1.29 (creatinine, pH 7).

### *P. piersonii* successfully forms dual species biofilm with *E. coli*

**Fig. 7:**
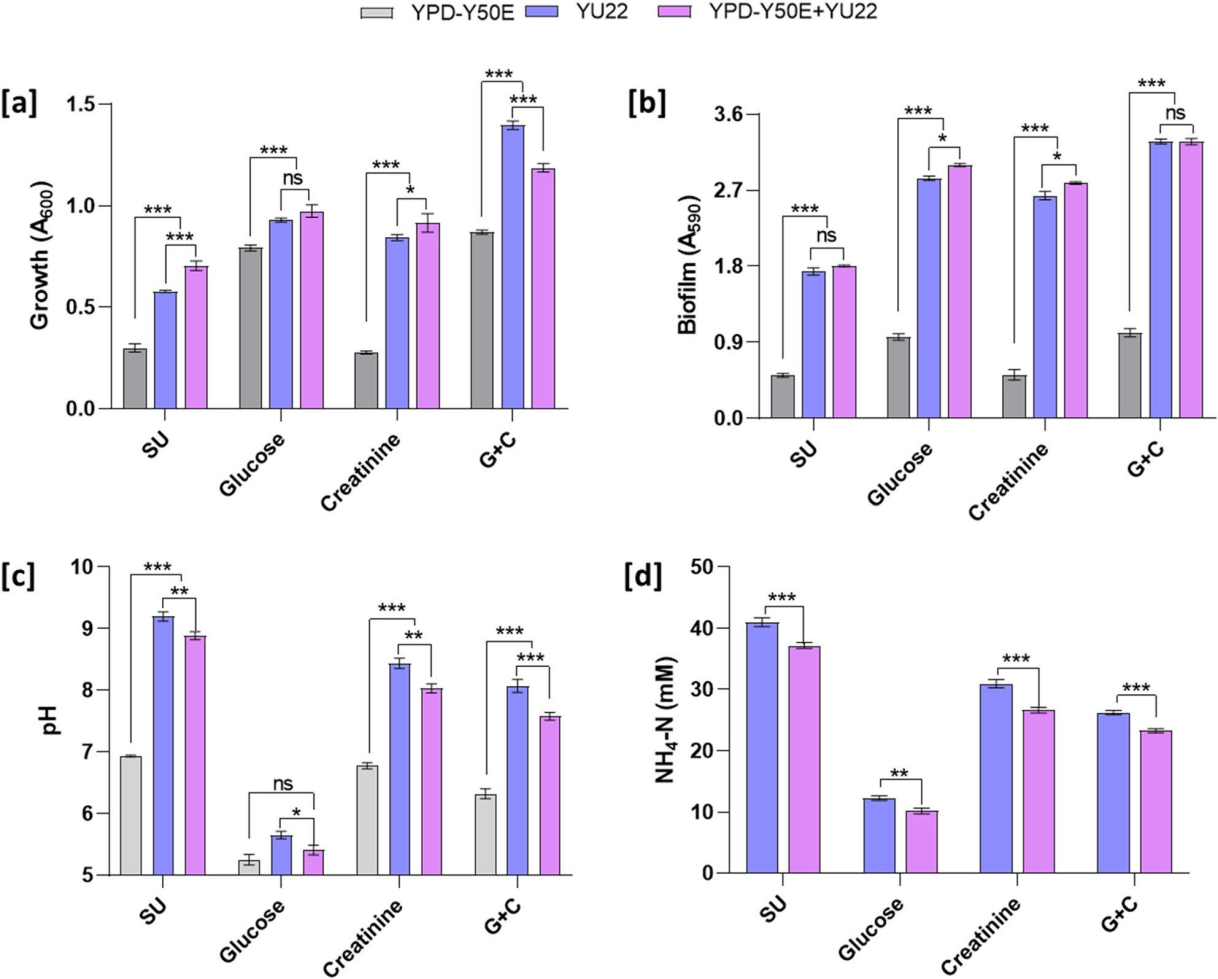
Interaction of *P. piersonii* with uropathogenic *E. coli* in a dual-species co-culture system grown in synthetic urine supplemented with glucose, creatinine, glucose (19 mM) with creatinine (53 mM) (G + C). Growth (A_600_) in the mono- and dual- species culture of *P. piersonii* and *E. coli* at 24 h (**a**), biofilm formation (**b**), pH changes (**c**), and NH_4_-levels (**d**). Data represent mean ± SD (*n* = 3). ns, *, ** and *** represent the significance of the difference at *p* > 0.05, *p* < 0.05, *p* < 0.01, and *p* < 0.001, respectively. ns; not significant.

To check the coexistence of *P. piersonii* with other uropathogens, it was co-cultured with *E. coli* under different nutritional conditions. *E. coli* YPD-Y50E was able to grow in the synthetic urine media as mono-species culture under control conditions (A_600_ 0.30 ± 0.02). Glucose in synthetic urine media promoted the *E. coli* growth, while, creatinine did not promote the growth significantly. The growth in dual-species culture was significantly higher than the *E. coli* mono-species culture in all the conditions. *P. piersonii*, in the glucose supplemented media showed no significant difference in the growth compared to the dual-species culture (Fig. 7a). In glucose + creatinine (G+C) supplemented media, the *P. piersonii* mono-species growth was higher compared to the dual-species culture. *P. piersonii* formed significantly higher biofilm in all the nutritional conditions compared to the *E. coli* strain. However, the biofilm intensity was similar to *P. piersonii* biofilm with creatinine and glucose combined growth media (*p* > 0.05) (Fig. 7b).

Similar to *P. piersonii*, in *E. coli* significant decrease in pH was observed from the initial pH of 6.8 under glucose containing media to pH 5.3 (Fig. 7c). The *E. coli* strain was urease negative and did not show urea conversion into NH_4_-N. The NH_4_-N levels in the dual-species cultures remained significantly lower than the *P. piersonii* mono-species culture (*p* < 0.01) (Fig. 7 d).

### Relative cell biomass of the bacteria in dual species biofilm

**Fig. 8:**
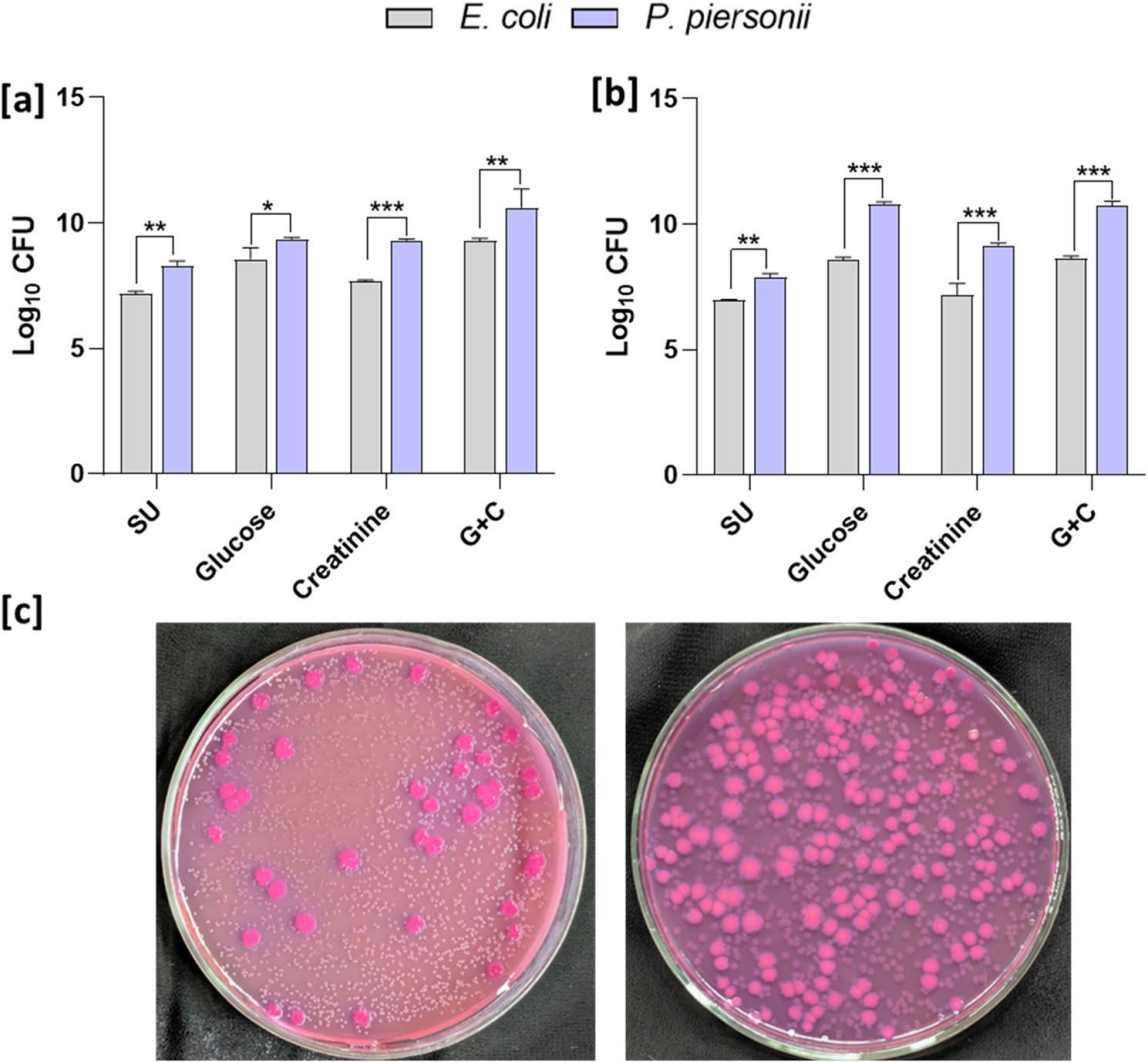
Dual species biofilm biomass (**a**) and planktonic biomass (**b**) of *P. piersonii* and *E. coli* grown in synthetic urine supplemented with glucose, creatinine, and a combination of glucose and creatinine (G + C). Representative images showing the dominance of *P. piersonii* colonies on MacConkey agar (**c**). Data represent mean ± SD (*n* = 3). *, **, and *** indicate significant differences at *p* < 0.05, *p* < 0.01, and *p* < 0.001, respectively.

Within the dual-species biofilm, the viable cell counts of individual bacteria varied under different nutritional conditions (Fig. 8a). The lowest cell biomass was seen in the synthetic urine control media where the log_10_ CFU of *E. coli* was 7.18 ± 0.01, and *P. piersonii* maintained a cell biomass of 8.28 ± 0.20 log_10_ CFU. In glucose and creatinine containing media, the biofilm cell viability was significantly higher with log_10_ CFU of 9.28 ± 0.11 and 10.61 ± 0.07 for *E. coli*, and *P. piersonii* respectively. In all conditions, *P. piersonii* cells were significantly more than *E. coli* (*p* < 0.05). Similarly, the planktonic cell biomass in the dual species culture of *E. coli* ranged from log_10_ CFU 6.96 ± 0.04 to 8.64 ± 0.10 (Fig. 8b). Both *E. coli* and *P. piersonii* biofilm biomass showed no significant difference between glucose and G + C media (*p* > 0.05). Representative images showing the dominance of *P. piersonii* in the biofilm biomass is illustrated in Fig. 8c. In the MacConkey agar the *E. coli* formed pink large colonies and *P. piersonii* formed small creamish white colonies.

## Discussion

The metabolic flexibility of uropathogens in the hostile urinary environment enables them to survive in urine or intracellularly within urothelial cells. In this study, we demonstrated that the urine-derived *P. piersonii* strain YU22 has tolerance to urea and is able to grow and form biofilm using urea as a sole carbon and nitrogen source in a broader range of urea concentrations up to 420 mM. In our previous study, we reported the survival ability of *P. piersonii* at the urea concentrations up to 10 mM [7]. This study demonstrates that *P. piersonii* may efficiently utilize urea, mediated by the UC-AH system and suggest its advantage for colonization in the urinary tract with limited nutritional supply. Many uropathogens have the ability to tolerate urea concentrations within the physiological urinary range (up to 320 mM) [38]. Generally, uropathogens use the enzyme urease for ureolytic activity and also have varied urea tolerance range [27, 39–41]. In many pathogens, UAL functions as a virulence trait; for example, in *Candida albicans*, it enables lethal systemic infections in immunocompromised patients [42, 43]. In *Granulibacter bethesdensis*, UAL mediated urea degradation promotes survival within macrophages and neutrophils, leading to persistent infections in individuals with chronic granulomatous disease [42]. More broadly, ureolytic activity helps many bacteria counteract acidic environments by alkalizing their surroundings. While urease has been extensively studied, the alternative ureolytic enzymes UC and AH remain poorly characterized, particularly in uropathogens [43]. In *G. bethesdensis*, the UC-AH pathway supports intracellular survival [44]. Within *Pantoea*, this system has been reported only in *P. ananatis*, which is associated with opportunistic human infections [45]. Notably, *P. agglomerans*, frequently isolated from human urine, exhibits a urease-negative phenotype [46]. 

The presence of glucose, creatinine, and albumin are commonly seen in patients with underlying metabolic and renal diseases. Our results demonstrated that in the synthetic urine the availability of these metabolites significantly favoured growth, biofilm formation, and associated virulence factors. Swarming motility and siderophore production contribute to virulence during UTI by facilitating the ascension and dissemination of bacteria within the urinary tract, allowing them to evade host immune responses and spread to new niches [47, 48]. *P. piersonii*, like many uropathogens, produces the siderophore and shows growth specific increase in its levels. These adaptive mechanisms strengthen the bacterial ability to colonize and persist in the urinary tract. Among the nutrients glucose enhanced the planktonic and biofilm mode of growth significantly. Genomic analysis of *P. piersonii* have revealed the presence of a gene encoding the cyclic adenosine monophosphate (cAMP) receptor protein (RTY57153), a known regulator involved in carbon catabolite repression. This regulatory mechanism can facilitate the preferential utilization of glucose as a primary carbon source, thereby decreasing the dependency on urea as a primary nutritional source when both are present [49]. Elevated urinary glucose levels, like glycosuria, create a nutrient-rich environment conducive to bacterial growth, which has been linked to increased incidence of UTI caused by many uropathogens [50, 51]. Supplementation of creatinine also favored the growth in *P*. *perisonii*. Some bacteria metabolize creatinine through pathways mediated by enzymes such as creatinine deaminase (in *Pseudomonas* spp. and *Klebsiella* spp.), cytosine deaminase (*Serratia* spp., *Providencia* spp., and *E. coli*), creatinine amidohydrolase (*K. pneumoniae* and *Mycobacterium tuberculosis*), and creatine amidohydrolase (*K. pneumoniae* and *Streptococcus pneumoniae*). In most bacterial species, creatinine metabolism involves its enzymatic conversion to creatine by creatininase, followed by further degradation to sarcosine and urea by creatinase. Subsequently, sarcosine is broken down to glycine and formaldehyde via sarcosine oxidase (SoxBDAG) or to methylamine via sarcosine reductase or glycine reductase [52]. However, exact mechanism by which *P. piersonii* utilizes creatinine requires further investigations. 

We also observed that presence of albumin in the synthetic urine media promoted growth and biofilm formation with enhanced swarming motility in *P. piersonii* strain YU22 compared to un-supplemented media. Albumin has been earlier reported to promote *S. aureus* infection by enhancing growth and strong biofilm formation by upregulating agr QS expression [38, 53]. However, albumin is not favorable for all the uropathogens, for example in *P. aeruginosa* albumin significantly reduce biofilm formation and downregulate quorum sensing [53, 54]. Our investigation on combination of metabolites in the synthetic urine also showed presence of additional metabolite enhanced the growth. Addition of creatinine with a lower glucose concentration or otherwise could result in higher increase in growth relative to their respective single scenarios. However, at higher concentrations the additional nutrition has slightly lesser additive effect. This may be attributed to the ability of *P. piersonii* to utilize both creatinine and glucose for its growth. Albumin supplied with all tested concentrations of glucose or creatinine showed increase in growth by 25% to 53%.

The changes in media pH varied under these conditions; though *P. piersonii* grown in glucose supplemented synthetic urine reduced the media pH, while, presence of creatinine or albumin increased the media pH at 24 h. The ureolytic mechanism is employed by many pathogens for the pH neutralization under acid stress. The results from our experiments under varying pH and different nutritional conditions demonstrated higher survival and growth at neutral pH. However, in the presence of glucose alkaline pH environment showed higher growth. Under acidic pH, creatinine supported the highest growth compared to glucose; however, the ureolytic activity was lower compared to the control with slight increase in media pH. On the other hand, *P. piersonii* further reduced the medium pH to the acidic range in glucose supplemented media across all initial pH conditions. Albumin supported ureolytic activity in all pH levels by further increasing the media pH. Interestingly, under control conditions in the absence of additional nutrients, the ureolytic activity was able to neutralize the acidic environment and further increased the media pH when grown in pH 8. In synthetic urine (control) at pH 5, ureolytic activity was higher than other pH conditions, likely representing a stress response to neutralizing acidity, as we observed the highest ΔpH. For instance, in *S. aureus*, urease-mediated urea hydrolysis increases extracellular pH and ammonium levels to maintain pH homeostasis in acidic host niches such as the kidney and skin, thereby enhancing bacterial persistence by improving fitness in the low-pH, urea-rich renal environment [55, 56]. Similarly, a report showed higher production of NH_4_-N in *E. coli* at acidic pH to maintain pH homeostasis [27]. Pathogens such as *Streptococcus salivarius*, *Helicobacter pylori*, and *Yersinia enterocolitica* exhibit higher urease activity in acidic environments, with peak ammonia production occurring at acidic pH [57–59]. These results demonstrate that the regulation of ureolytic activity of *P. piersonii* is complex with the ability to adapt to environmental conditions based on the nutrients for its survival in prevailing urinary conditions. 

The results of co-culture experiments demonstrated the ability of *P. piersonii* to co-exist in poly microbial communities with *E. coli* and important uropathogen. The nutritional status of the growth media played an important role in the co-existence; in all conditions, *P. piersonii* showed relative dominance over *E. coli* based on the CFU data. One of the reasons as the tested *E. coli* strain was urease negative *P. piersonii* would have benefited from the advantage of its ureolysis system. Further, *P. piersonii*, similar to *E. coli*, also has a preference for glucose; the competition for preferential nutrients may provide slight advantages for organisms with more nutritional flexibility under complex nutritional environments. As there are very limited reports on *Pantoea* species and their role as uropathogens *P. piersonii* strain YU22 may serve as a model organism for further studies with other uropathogens. The modulation of growth, biofilm formation, and virulence factor production under varying nutritional and environmental conditions warrants further investigation, particularly in relation to both intra- and interspecies quorum sensing systems. The presence of an autoinducer-2 (AI-2) mediated *luxS* quorum-sensing system has been identified from the genome data; however, its role in the regulation of biofilm formation and virulence factors is yet to be established.

## Conclusion

The ability of *P. piersonii* to differentially regulate its metabolic and biofilm-related mechanisms based on urine composition and pH may confer a survival advantage under diverse urinary conditions. The findings of this study highlight that *P. piersonii* YU22 exhibits metabolic adaptations to urine environment including metabolic diseases associated with comorbidities, underlining its potential as an opportunistic pathogen. Its ability to form biofilms with other uropathogens such as *E. coli* warrants further studies as it may pose challenges in managing the UTI. Together, the results suggest that *P. piersonii* strain YU22 employs a complex, nutrient-dependent strategy to modulate ureolytic activity and sustain survival under extreme conditions. Further validation using targeted molecular and protein expression studies is necessary to substantiate these regulatory mechanisms.

## Supplementary Information

Supplementary Material 1.

## Data Availability

Data used and/or analysed during the current study are available on request.
