# Supplementary material for: Effect of metabolites on the survival and biofilm formation of Pantoea piersonii (Basionym: Kalamiella piersonii) in synthetic urine media
Source: BMC Microbiol. 2026 Jan 20;26:182. doi: 10.1186/s12866-025-04684-z (PMC12951989; doi:10.1186/s12866-025-04684-z)
Supplement: Supplementary file 1 — Supplementary Material 1. [file 12866_2025_4684_MOESM1_ESM.docx]

**Effect of Metabolites on the Survival and Biofilm Formation of *Pantoea piersonii* (Basionym: *Kalamiella piersonii*) in Synthetic Urine Media**

Yuvarajan Subramaniyan^1^, K S Megha ^1^, Adithyan K^1^, Rajendu R Nair^1^, M Mujeeburahiman^2^, Blessy M Baby^1^, Pallavi Poojarira G^1^, Punchappady Devasya Rekha^*1^

1. Division of Microbiology and Biotechnology, Yenepoya Research Centre, Yenepoya (Deemed to be University), University Road, Deralakatte, Mangalore - 575018, India.
2. Department of Urology, Yenepoya Medical College and Hospital, Yenepoya (Deemed to be University), University Road, Deralakatte, Mangalore -575018, India.

*Corresponding author:

Dr. P.D. Rekha

Professor, Division of Microbiology and Biotechnology

Yenepoya Research Centre, Yenepoya (Deemed to be University)

University Road, Deralakatte, Mangalore - 575018, India.

Phone: +91 9741501821; Email: [rekhapd@yenepoya.edu.in](mailto:rekhapd@yenepoya.edu.in)

**ORCID**

Punchappady Devasya Rekha: <https://orcid.org/0000-0002-9187-6395>

Yuvarajan Subramaniyan: <https://orcid.org/0000-0003-1172-0938>

Mujeeburahiman M: <https://orcid.org/0009-0001-6653-5791>

Blessy M Baby: <https://orcid.org/0009-0009-8032-9052>

K S Megha: <https://orcid.org/0009-0007-5967-9512>

**Table S1.** Composition of the developed M9-urea (M9U) medium

| **Sl.no** | **Components** | **Concentration** |
| --- | --- | --- |
| 1 | NaCl | 8.6 mM |
| 2 | KH_2_PO_4_ | 14.7 mM |
| 3 | MgSO_4_ | 1.0 mM |
| 4 | NiCl_2_ | 0.34 μM |
| 5 | CaCl_2_ | 0.1 mM |
| 6 | ZnSO_4_ | 50 μM |
| 7 | FeCl_2_ | 20 μM |
| 9 | Urea* | 10 - 420 mM |

*Urea concertation was used in the range of 10 to 420 mM to study its effect on the bacterial growth.

All components were aseptically dissolved in sterile Milli-Q water. All M9U components except urea, FeCl_2,_ and ZnSO_4_ were mixed and the medium was autoclaved. Urea, FeCl_2_ and ZnSO_4_ were sterile‐filtered and added from a stock solution to achieve the desired concentration aseptically after autoclaving.

**Table S2.** Details of primers used in qRT-PCR analysis of the expression of genes encoding urea carboxylase (UC) and allophanate hydrolase (AH)

| **Primer** | **Sequence (5'→3')** | **Product size (bp)** | thermal cycling conditions |
| --- | --- | --- | --- |
| UC_F | ctatgacagcctgcacctca | 208 | Initial denaturation at 95 °C for 3 min, followed by 40 cycles of 95 °C for 10 s (denaturation) and 56 °C for 30 s (annealing). Melt-curve analysis was performed from 65-95 °C |
| UC_R | atcgacgttgtttggtagcc |  |  |
| AH_F | atacctgcgagccgaactta | 165 |  |
| AH_R | gcaaggttggtgaagttggt |  |  |

**Fig. S1**. Effect of urea levels on the ureolytic activity of *P. piersonii* YU22 over 72 h growth. The NH_4_-N levels were measured under 160 mM and 420 mM of urea. Data represent mean ± SD (n = 3). ns - not significant *p* > 0.05. ns.


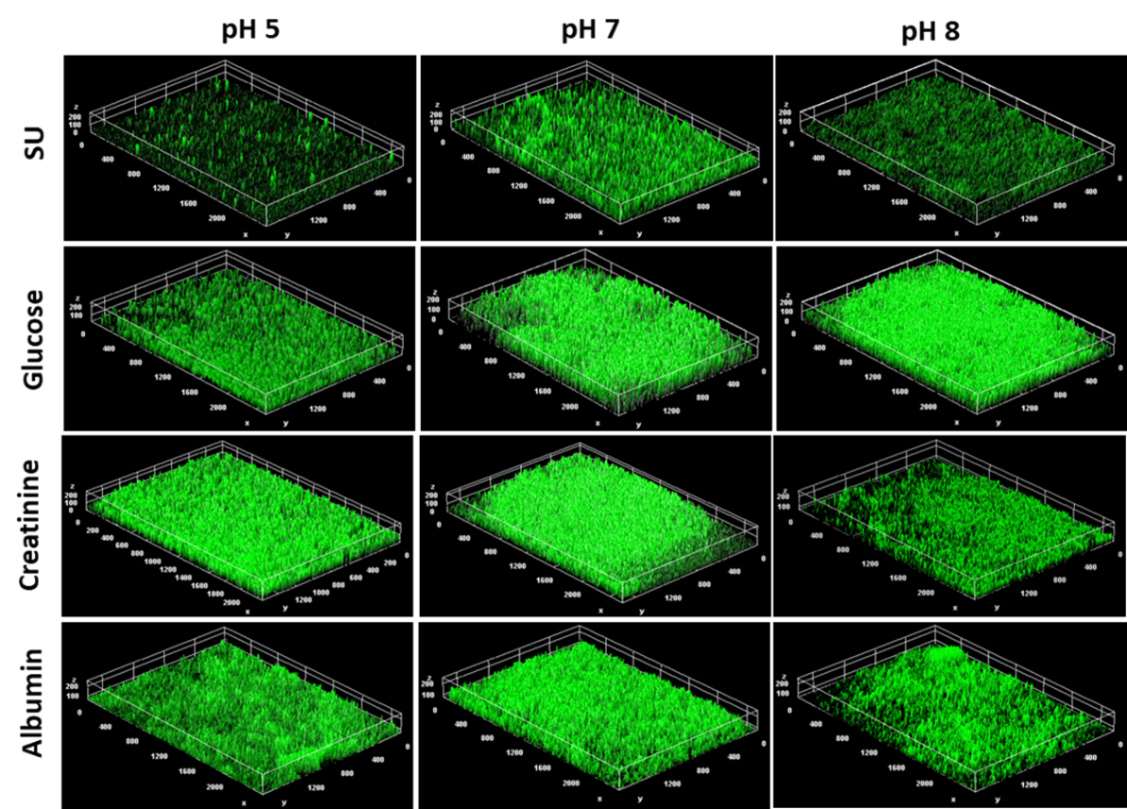


**Fig. S2.** **Influence of pH on biofilm formation in *P. piersonii* YU22 under different nutritional conditions at 24 h.** Representative fluorescence Z-Stack images are shown for illustration of the acridine-orange-stained biofilms developed on glass coupons.

**Fig. S3**. Influence of pH on ureolytic activity mediated ammonia release by *P. piersonii* YU22 under different nutritional conditions. Data represent mean ± SD (n = 3). * and *** represent the significance of the difference at *p* < 0.05 and *p* < 0.001, respectively.

**Table S3:** Effect of albumin and its combination with glucose and creatinine supplemented synthetic urine on growth, biofilm formation, pH and NH_4_-N levels of *P. piersonii.*

| **Treatments** | **Code** | **Growth (A_600_)** | ***Biofilm (*A_590_)** | ***pH*** | **NH_4_-N (mM)** |
| --- | --- | --- | --- | --- | --- |
| Glucose | G1 | 0.65 ± 0.01 | 1.82 ± 0.02 | 7.58 ± 0.07 | 19.11 ± 0.67 |
|  | G2 | 0.90 ± 0.01 | 2.88 ± 0.04 | 5.80 ± 0.03 | 13.44 ± 0.56 |
|  | G3 | 1.22 ± 0.02 | 3.08 ± 0.02 | 4.96 ± 0.10 | 7.83 ± 0.81 |
| Creatinine | C1 | 0.58 ± 0.01 | 1.71 ± 0.02 | 8.04 ± 0.09 | 24.01 ± 0.63 |
|  | C2 | 0.87 ± 0.01 | 2.62 ± 0.02 | 8.35 ± 0.06 | 32.19 ± 1.13 |
|  | C3 | 1.02 ± 0.01 | 2.87 ± 0.08 | 8.70 ± 0.06 | 39.81 ± 0.62 |
| Albumin | A | 0.79 ± 0.01 | 2.53 ± 0.08 | 8.21 ± 0.09 | 35.9 ± 1.1 |
| Glucose + Albumin | G1+A | 0.89 ± 0.03 | 3.06 ± 0.21 | 7.92 ± 0.03 | 21.1 ± 0.04 |
|  | G2+A | 1.28 ± 0.01 | 3.46 ± 0.01 | 7.67 ± 0.07 | 18.0 ± 1.1 |
|  | G3+A | 1.53 ± 0.03 | 3.47 ± 0.03 | 7.31 ± 0.05 | 12.8 ± 0.4 |
| Creatinine + Albumin | C1+A | 0.89 ± 0.02 | 1.68 ± 0.47 | 8.33 ± 0.09 | 38.5 ± 0.5 |
|  | C2+A | 1.23 ± 0.04 | 2.92 ± 0.05 | 8.77 ± 0.09 | 41.1 ± 1.9 |
|  | C3+A | 1.41 ± 0.02 | 3.28 ± 0.06 | 9.06 ± 0.05 | 45.2 ± 0.4 |

Different glucose and creatinine concentrations supplemented in synthetic urine were used. G1, G2 and G3 respectively are glucose used at 5 mM, 19 mM and 35 mM. C1-10 mM Creatinine, C2- 53 mM Creatinine, C3-100 mM Creatinine, A- 300 mg/L Albumin. Growth was measured as A600 nm, and biofilm was quantified using crystal violet staining as A_590_ nm, pH changes were measured using a digital pH meter.
